# A Fiber Optic Interferometric Sensor Platform for Determining Gas Diffusivity in Zeolite Films

**DOI:** 10.3390/s18041090

**Published:** 2018-04-04

**Authors:** Ruidong Yang, Zhi Xu, Shixuan Zeng, Wenheng Jing, Adam Trontz, Junhang Dong

**Affiliations:** 1 Department of Chemical and Environmental Engineering, University of Cincinnati, Cincinnati, OH 45221, USA; yangrg@mail.uc.edu (R.Y.); zhi.xu@eng.ox.ac.uk (Z.X.); zengsn@mail.uc.edu (S.Z.); trontzam@mail.uc.edu (A.T.); 2 State Key Laboratory of Materials-Oriented Chemical Engineering, Nanjing Tech University, 5 Xinmofan Road, Nanjing 210009, China; jingwh@njtech.edu.cn

**Keywords:** fiber optic interferometer, zeolite film, optical sensing, molecular diffusivity

## Abstract

Fiber optic interferometer (FOI) sensors have been fabricated by directly growing pure-silica MFI-type zeolite (i.e., silicalite) films on straight-cut endfaces of single-mode communication optical fibers. The FOI sensor has been demonstrated for determining molecular diffusivity in the zeolite by monitoring the temporal response of light interference from the zeolite film during the dynamic process of gas adsorption. The optical thickness of the zeolite film depends on the amount of gas adsorption that causes the light interference to shift upon loading molecules into the zeolitic channels. Thus, the time-dependence of the optical signal reflected from the coated zeolite film can represent the adsorption uptake curve, which allows computation of the diffusivity using models derived from the Fick’s Law equations. In this study, the diffusivity of isobutane in silicalite has been determined by the new FOI sensing method, and the results are in good agreement with literature values obtained by various conventional macroscopic techniques. The FOI sensor platform, because of its robustness and small size, could be useful for studying molecular diffusion in zeolitic materials under conditions that are inaccessible to the existing techniques.

## 1. Introduction

Zeolitic materials, including the crystalline microporous aluminosilicates and their structural analogs of pure silica, borosilicates and aluminophosphates, are versatile materials for developing advanced catalysts, adsorbents, ion exchangers, separation membranes, and membrane reactors with board applications in chemical and energy industries and environmental management systems. The molecular transport diffusivity in the zeolitic pores plays a key role in determining the rates and selectivity of separation or reaction [1]. Thus, the measurement of molecular or ionic diffusivity in zeolitic materials has been a constant focus in the research community [2,3]. Although substantial progress has been made in experimental measurement and theoretical computation of molecular diffusivity in zeolites, uncertainties and large discrepancies exist among diffusivity values measured by different methods because of various limitations associated with the current measurement techniques. These limitations may include the difficulty to exclude external mass transfer resistance and effect of heat of adsorption in the macroscopic measurement methods, such as the chromatography (CHG) or microbalance monitored breakthrough curve approaches, the zero length column (ZLC) technique, and the transient membrane (permeation) (MBR) method, and the inefficiency to operate continuously under non-equilibrium conditions for microscopic techniques, such as pulse field gradient (PFG) nuclear magnetic resonance (NMR) measurement [4,5,6]. In addition, restrictions on operation conditions for the existing microscopic and macroscopic techniques have caused difficulties in examining the diffusivity in more practically meaningful temperature and pressure ranges and chemical environments.

Zeolite crystals possess a number of optical properties that are altered by inclusion of adsorbate molecules into the zeolitic pores. The changes in optical properties of the zeolite crystals induced by molecular adsorption can be detected by appropriate spectroscopic methods such as infrared (IR) absorption spectroscopy, laser refractometry , interference microscopy, IR microscopic imaging, and Raman scattering [7,8,9,10,11,12]. These offer unique opportunities for developing optical means to investigate the transport, catalytic, and thermodynamic properties of the adsorbate–zeolite interactions. The optical properties change instantly with the variation of adsorbate loading in the zeolite. Thus, the temporal optical response of the zeolite during the dynamic process of adsorption can be utilized for diffusivity measurements.

Here, we demonstrate a novel zeolite thin film-coated fiber optic interferometer (FOI) sensor platform for determining gas diffusivity in zeolites. The FOI sensor head is of micrometer size where the very small amount of zeolite thin film sample can effectively mitigate the influence of mass transport resistance in sample beds employed by traditional macroscopic methods. Also, the minimal amount of heat of adsorption in the zeolite thin film can be removed timely by fast heat conduction through the intimately connected dense fiber. The diffusivity measurement is carried out by highly sensitive real-time monitoring of the optical interference signal reflected from the zeolite film during the dynamic process of molecular adsorption.

## 2. Experimental Section

### 2.1. Sensor Structure and Operation Principle

**Figure 1:**
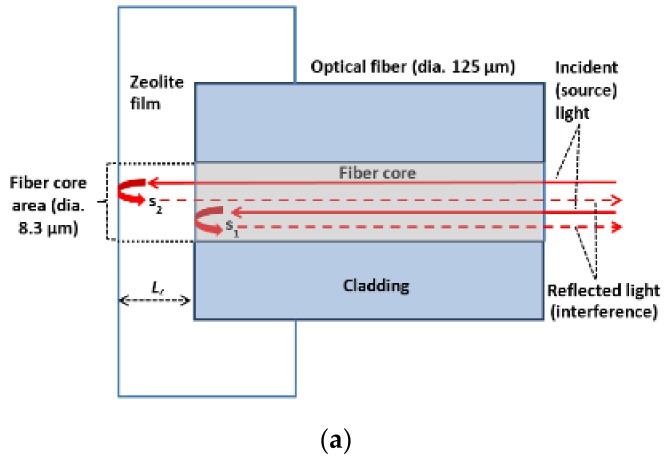

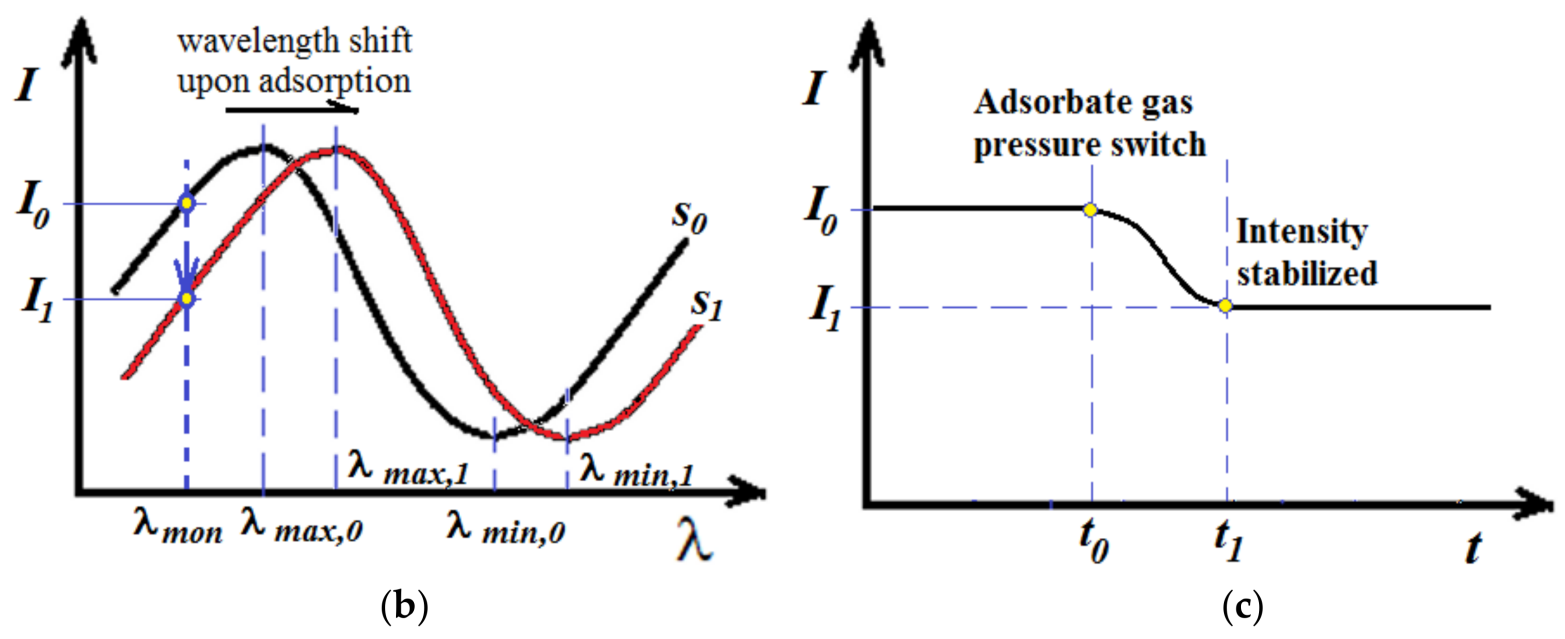
Schematics showing (**a**) the zeolite film-coated fiber optic interferometer (FOI) sensor head; (**b**) illustration of the reflected light interference spectrum shift upon adsorption; and (**c**) reflection intensity (I; signal S) as a function of time at a fixed wavelength during the dynamic process of adsorption.

The zeolite film-coated FOI sensor is schematically depicted in Figure 1. The sensor head is a straight-cut endface of an optical fiber coated with a polycrystalline thin film of the zeolite to be examined. The present study employed a regular communication optical fiber, which had a 125-μm-diameter pure silica cladding and an 8.3-μm-diameter Ge-doped silica core (Corning Inc., Corning, NY, USA). In operation, the source light is sent through the fiber core to interact with the zeolite film covering the core area (diameter = 8.3 μm). Two reflected lights (*s*_1_ and *s*_2_) are generated at the fiber/zeolite and zeolite/gas interfaces, respectively, which interfere and transmit back through the fiber core to be recoded. The zeolite thin film coated on the cleaved fiber endface can be treated as a low finesse intrinsic Fabry-Pérot interferometer (FPI), where the two-beam interference model applies [8]. The inference signal (S) is described by the following sinusoidal waveform equation [13],

(1) $$S=r_{1}^{2}+\;\alpha r_{2}^{2}+2\alpha r_{1}r_{2}cos\left(\frac{4\pi L_{z}n_{z}}{\lambda }+\varphi_{0}\right)$$

where, $r_{1}$ and $r_{2}$ are the amplitude reflection coefficients of the fiber/zeolite and zeolite/environment interfaces, respectively; $\varphi_{0}$ is the phase of the interference; $L_{z}$ is the physical thickness of the zeolite film; $n_{z}$ is the refractive index of the zeolite; λ is the light wavelength; and α is the lumped amplitude loss coefficient at the optical interfaces.

Because both the *L_z_* and *n_z_*, and hence the optical thickness *L_z_n_z_*, change when varying the amount of molecules loaded in the zeolitic pores, the interference spectrum (i.e., interferogram) depicted in Figure 1b exhibits wavelength shift upon adsorbing molecules into the zeolite film [8]. When the sensor head is put in an environment of adsorbate gas, the interference signal (S) changes with time and stabilizes when equilibrium is reached between the zeolite film and the environmental adsorbates [14,15]. Thus, the molecular diffusivity can be determined by monitoring the temporal evolution of the optical interference signal from the zeolite film-coated FOI during the dynamic process of adsorption when the relationship between the interference and amount of adsorption is established for the adsorbate molecule. More specifically, the dynamic curve of molecular adsorption represented by the time-dependence of optical signal S can be utilized to calculate the transport diffusivity *D*(*C*) by various models derived from the Fick’s law equations [6].

However, scanning and recording the entire interferogram by common instruments such as optical spectrum analyzer (OSA) and tunable leaser require relatively long time that gives insufficient time resolution for studying molecules of large diffusivity. To achieve desirable time resolution, single wavelength reflectance monitoring can be employed because existing instruments can offer data acquisition speed up to 1 MHz if necessary. As illustrated in Figure 1c, a specific wavelength can be identified where monotonic increase or decrease of the intensity is attainable over the entire equilibrating process for convenient monitoring and data processing. Thus, the FOI sensor platform can be used to study the gas diffusivity in the coated zeolite film by two optical measurements performed simultaneously using tunable laser: (1) measurement of interference spectrum as a function of adsorbate concentration under equilibrium conditions for establishing the relationship between reflectance and adsorbate concentration; and (2) measurement of single wavelength reflectance as a function of time during the dynamic process of adsorption that provides the time-dependence of adsorbate concentration in zeolite. 

### 2.2. Sensor Fabrication

**Figure 2:**
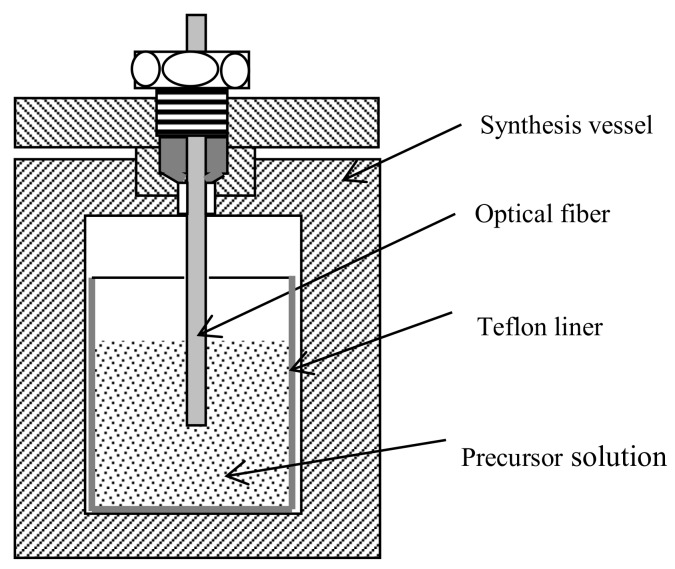
Schematic showing the cleaved fiber end mounted in the synthesis vessel.

The pure silica MFI-type zeolite (i.e., silicalite) film was directly grown on the cleaved endface of a single mode optical fiber, which had a 125-μm-diameter pure silica cladding and an 8.3-μm-diameter Germanium-doped core (SMF28^TM^, Corning Co., Corning, NY, USA). The detailed synthesis procedure was similar to that described in our previous publications [8,15]. The silicalite synthesis solution was prepared by mixing 30 mL deionized (D.I.) water, 5.65 mL 1 M tetrapropyl ammonium hydroxide (TPAOH; Sigma, Ronkonkoma, NY, USA) solution, and 10.2 mL tetraethyl orthosilicate (TEOS; Sigma, St. Louis, MO, USA). The TPAOH was used as structural directing agent (SDA) for the MFI-type zeolites. An amount of 5 mL of the clear solution was transferred into a Teflon-lined stainless steel synthesis vessel. The cleaved optical fiber end was mounted into the synthesis vessel with the endface facing downwards and about 1 cm of the end segment immersed in the synthesis solution, as schematically shown in Figure 2. The synthesis vessel was then moved into an oven preheated to 180 °C where the in situ hydrothermal crystallization reaction was conducted at 180 °C for 4 h. After the hydrothermal treatment, the zeolite-coated fiber end was rinsed with D.I. water and further cleaned in an ultrasonic bath (Cole Parmer 8890, Vernon Hills, IL, USA) for 5 min. The zeolite particles formed in the liquid phase during the film synthesis were collected for crystal structure and chemical characterizations and gas adsorption measurements. The above synthesis process was repeated for two more times to increase the thickness of the zeolite film so that a complete period of the sinusoidal interferogram can be generated for ease of data processing. The reflected interferogram quality depends on the smoothness of the zeolite film surface due to light scattering effects. To ensure a strong reflected optical signal, the outer surface of the as synthesized zeolite film was finely polished by an Ultra-Tech precision polishing system [16]. The zeolite film-coated fiber was dried at 80 °C in an oven and subsequently calcined at 550 °C in air for 4 h to remove the SDA molecules from the zeolite pores with both heating and cooling rates of 5 °C/min. The morphology and thickness of the zeolite film were observed by scanning electron microscopy (SEM) and the thickness of the zeolite film without adsorption (i.e., *L_z,_*_0_) was accurately determined from its interference spectrum as explained in the later sections. 

### 2.3. Apparatus and Measurement Procedure

**Figure 3:**
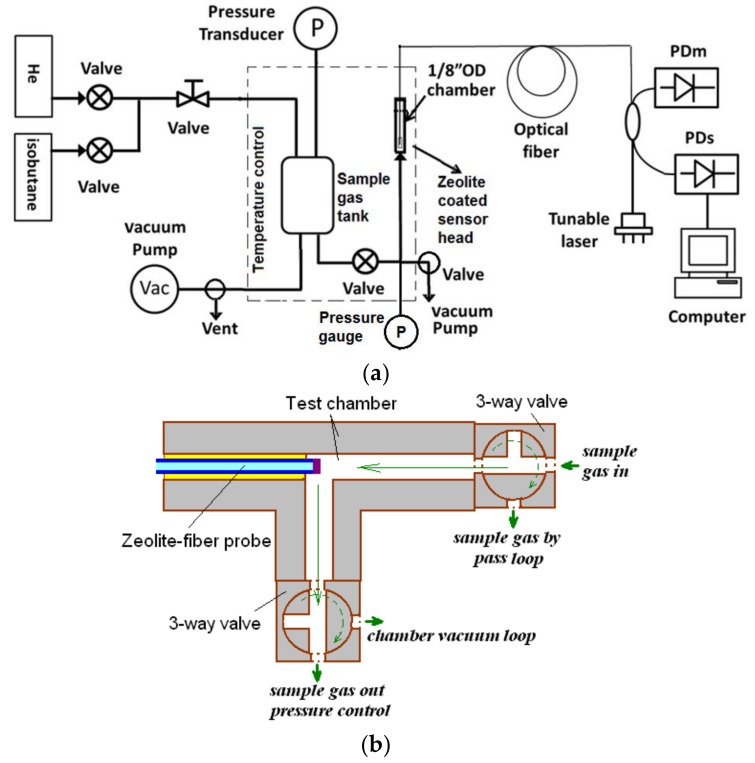
Schematics showing (**a**) the experimental system for FOI sensor operation; and (**b**) the FOI sensor head installation in the testing chamber.

The experimental apparatus for operating the zeolite film-coated FOI sensor is schematically shown in Figure 3. The FOI sensor was vertically inserted into a 1/8″ stainless steel Swagelok T-connector with the zeolite coated endface sensor head positioned in the center of the gas chamber of a very small volume (~0.076 cm^3^) in between two valves. The optical fiber was mounted into the system using silicon rubber septa seals (Supelco, Bellefonte, PA, USA). The source light was provided by a tunable laser equipped with a laser power detector (Agilent 8164A, Alpharetta, GA, USA) and a computer data acquisition system. The reflected interferogram was obtained by sweeping from 1510 to 1640 nm wavelength. A wavelength increment of 0.1 nm and a dwelling time of 1 s were used in scanning the entire interference spectrum. For monitoring the single wavelength response, the reflected light intensity was recorded at a 10-Hz frequency. The sensor chamber was connected to a gas tank with a volume of 150 cm^3^, which was large enough to neglect the pressure change when the valve was opened to connect it with the sensor chamber (0.076 cm^3^). The gas tank, sensor chamber, and connecting tubing and valves were rigorously degassed first by alternating the high purity helium (99.999%, Wright Bros. Inc., Cincinnati, OH, USA) purging and vacuuming 3–5 times and then by further vacuuming at 180–250 °C for 2 h using a dry vacuum pump (<2 Pa). The unit was then cooled down to a preset temperature for recording baseline interferogram under vacuum. The valve between the sensor chamber and the gas tank was then closed and the gas tank was charged with the sample gas of a given pressure, which was monitored by a precision pressure transducer. The sensor chamber was monitored by a precision digital pressure gauge as well to detect any internal leaking in the tubing line. The system was also modified by replacing the sample gas tank with direct connection to the sample gas flow when helium carrier gas was used. In this modified apparatus, direct connection of the sensor to sample gas flow with pre-determined adsorbate partial pressure (or volume fraction) allows high flow rate sweeping over the sensor head (in the unit shown in Figure 3b) to minimize surface convection resistance. The results of the modified unit were used to confirm that surface mass transport resistance was also negligible when sample gas comes from the gas tank.

The interferogram expressed by Equation (1) is influenced by the source light spectrum. Thus, for eliminating the source light modulation, the interferogram was normalized by a reference spectrum reflected from a cleaved fiber endface before splicing the sensor to the system [16]. The optical measurement of the dynamic adsorption processes used the following procedure: (1) the baseline interferogram was measured when the signal was stabilized after degassing without adsorbate gas in the sensor chamber; (2) measurement was then switched to monitoring single wavelength reflectance at a 10 Hz recording speed during which the sample gas was introduced by opening the valve located between the sample gas tank and sensor (or to the sample gas flow); (3) when the single wavelength reflectance was stabilized (i.e., adsorption reached equilibrium), the interferogram was scanned and recorded again; and (4) the adsorbate partial pressure in the flow was increased by a small increment and the operation procedure was repeated for staircase measurement. When the gas tank was used to measure FOI sensor response to a single step increase of *P_isob_* from 0 Pa to different values, complete degassing was performed for measurement of each gas pressure. The adsorbing gas pressure was varied for studying the effects of the adsorbate concentration in zeolite on the molecular diffusivity. Experiments were also performed at various temperatures to evaluate the apparent activation energy for molecular diffusion.

### 2.4. Determination of Optical Thickness of the Zeolite Film

The optical thickness of the zeolite film without gas adsorption ($L_{z,0}n_{z,0}$) was calculated from the baseline interferogram by Equation (2), which is derived from the trigonometric function in Equation (1). The calculation was based on the spectral positions of the adjacent interference peak $\lambda_{max,0}$ and valley $\lambda_{min,0}$ of the baseline interferogram. Thus, the actual physical thickness of the film can be determined when refractive index of zeolite is known, and vice versa. The silicalite has been previously determined to have a *n_z,_*_0_ of 1.3361 in the near-IR region used in this work [8]

(2) $$L_{z,0}n_{z,0}=\frac{1}{4}\left(\frac{1}{1/\lambda_{max,0}-1/\lambda_{min,0}}\right)\;$$

When the adsorbate concentration in zeolite changes from *C_k-_*_1_ to *C_k_* (*k* = 1, 2, …), the spectral positions of the interference peak ($\lambda_{max,k-1}$) and valley $(\lambda_{min,k-1}$) shift to $\lambda_{max,k}$ and $\lambda_{min,k}$, respectively, due to the change of optical thickness. The optical thicknesses at these two concentrations are related by Equation (3),

(3) $$L_{z,k}n_{z,k}=\frac{\lambda_{min,k}}{\lambda_{min,k-1}}L_{z,k-1}n_{z,k-1}$$

The shift of *λ_max,k_* and *λ_min,k_* as a function of adsorbate concentration (*C*) can be conveniently monitored during the staircase increases of *C* starting at reference state of *C* = 0. Thus, the FOI interferogram as a function of the gas adsorption level (*C*) can be readily processed by Equations (2) and (3) to provide the relationship between optical thickness (*L_z_n_z_*) and adsorbate concentration in the coated zeolite film. 

It is worth noting that when this method is used for measuring zeolite films of unknown *n_z,_*_0_ values, the following Equation (4) can be used to calculate the zeolite reflective index (*n_z_*) from the experimentally measured interferogram at any given conditions [8]. The model calculation is based on the intensities of the neighboring peak (*S_max_*) and valley (*S_min_*) that are expressed by the interference Equation (1) and the reflective index of the fiber core ($n_{f}$), which is 1.4682 for the Ge-doped silica fiber core used in this work (SMF28^TM^, Corning) and is usually available from the manufacturer, should a different fiber be used.

(4) $$n_{z}=\frac{2-(\sqrt{S_{\max}}+\sqrt{S_{\min}})}{2+(\sqrt{S_{\max}}+\sqrt{S_{\min}})}n_{f}$$

### 2.5. Determination of Diffusivity 

Isobutane was used as a model molecule for demonstration and validation of this diffusivity measurement method by the FOI sensor platform. In order to determine the relationship between zeolite optical response and adsorbate loading and to investigate the effect of adsorbate concertation on molecular diffusivity in the zeolite pores, adsorption isotherms were measured for isobutane by the Micromeritics ASAP 2020 unit (Micromeritics Instrument Corp., Norcross, GA, USA). The isotherms were determined at temperatures varying from 24 °C to 120 °C. The isobutane concentration in the zeolite was calculated based on the measured amount of adsorption and the silicalite density. The time-dependence of the FOI reflectance was translated into data of adsorbate concentration change with time during the dynamic adsorption process, which is also known as the uptake curve. Because the 8.3-μm-diameter zeolite thin film covering the fiber core area is much smaller than the entire zeolite film on the 125-μm-diameter endface, the model of 1-dimensional diffusion transport along the thickness of a slab is well-suited. Thus, under transient state of adsorption, the adsorbate concentration in the zeolite is described by the Fick’s second law equation [6]:

(5) $$\frac{\partial C}{\partial t}=\frac{\partial }{\partial x}\left[D(C)\left(\frac{\partial C}{\partial x}\right)\right]$$

where *D*(*C*) is the concentration-dependent diffusivity. For a very small concentration gradient created by a small increment of adsorbate gas pressure, the diffusivity can be treated as a constant and Equation (5) becomes [17]

(6) $$\frac{\partial C}{\partial t}=D(\bar{C})\frac{\partial^{2}C}{\partial x^{2}}$$

where $\bar{C}$ signifies the average of equilibrium concentrations before and after the small change of adsorbate gas pressure. Consequently, the concentration-dependence of the diffusivity can be obtained by a staircase measurement where constant diffusivity can be assumed for each small increment of environmental gas pressure. The Fick’s law equation is solved for the condition of constant surface concentration (C*), which is determined from adsorption isotherms, i.e., $C^{*}=C_{\infty }$, where $C_{\infty }$ is the equilibrium adsorbate concentration in zeolite at the applied gas pressure. Within the Henry’s region of isotherms where adsorbate concentration is sufficiently low (C $<0.5C_{max};$ $C_{max}$ is the maximum load), the following approximated solution can be used [2,9]

(7) $$\frac{C_{t}-C_{0}}{C_{\infty }-C_{0}}=\left(2/L_{z}\right)\sqrt{\frac{D\left(C\right)\cdot t}{\pi }}$$

where $C_{t}$ is the sorbate loading at time *t* after switching gas pressure; $C_{0}$ is the initial loading. With the experimentally determined relationship between the FOI sensor reflection intensity (S) and *C_∞_*, *C_t_* at any time can be obtained from the temporal response of single wavelength intensity for [$C_{t}-C_{0})/(C_{\infty }-C_{0})]<1.0$. The D(C) value at $\bar{C}=0.5(C_{0}+C_{t})$ is then calculated by Equation (7). In this work, the *t* values were read at intensity corresponding to $\left[\left(C_{t}-C_{0}\right)/(C_{\infty }-C_{0}\right)]$ values of around 0.75 rather than taking the equilibrating time because the later could involve large human errors in determining the full stabilization state.

## 3. Results and Discussion

### 3.1. Zeolite Film-Coated FOI Sensor

**Figure 4:**
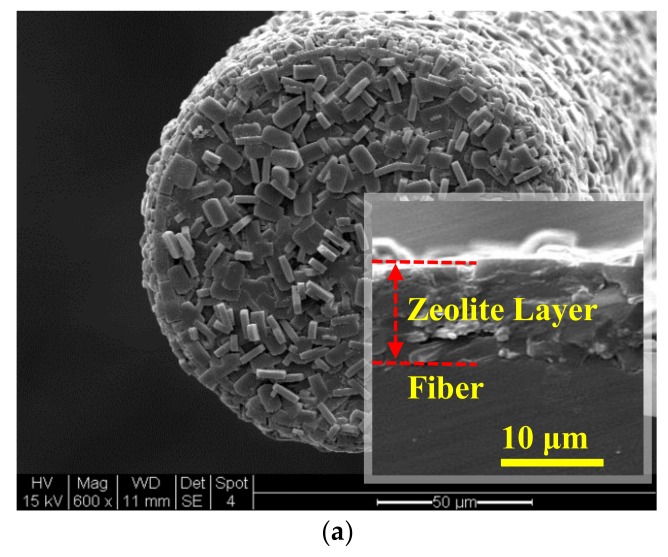

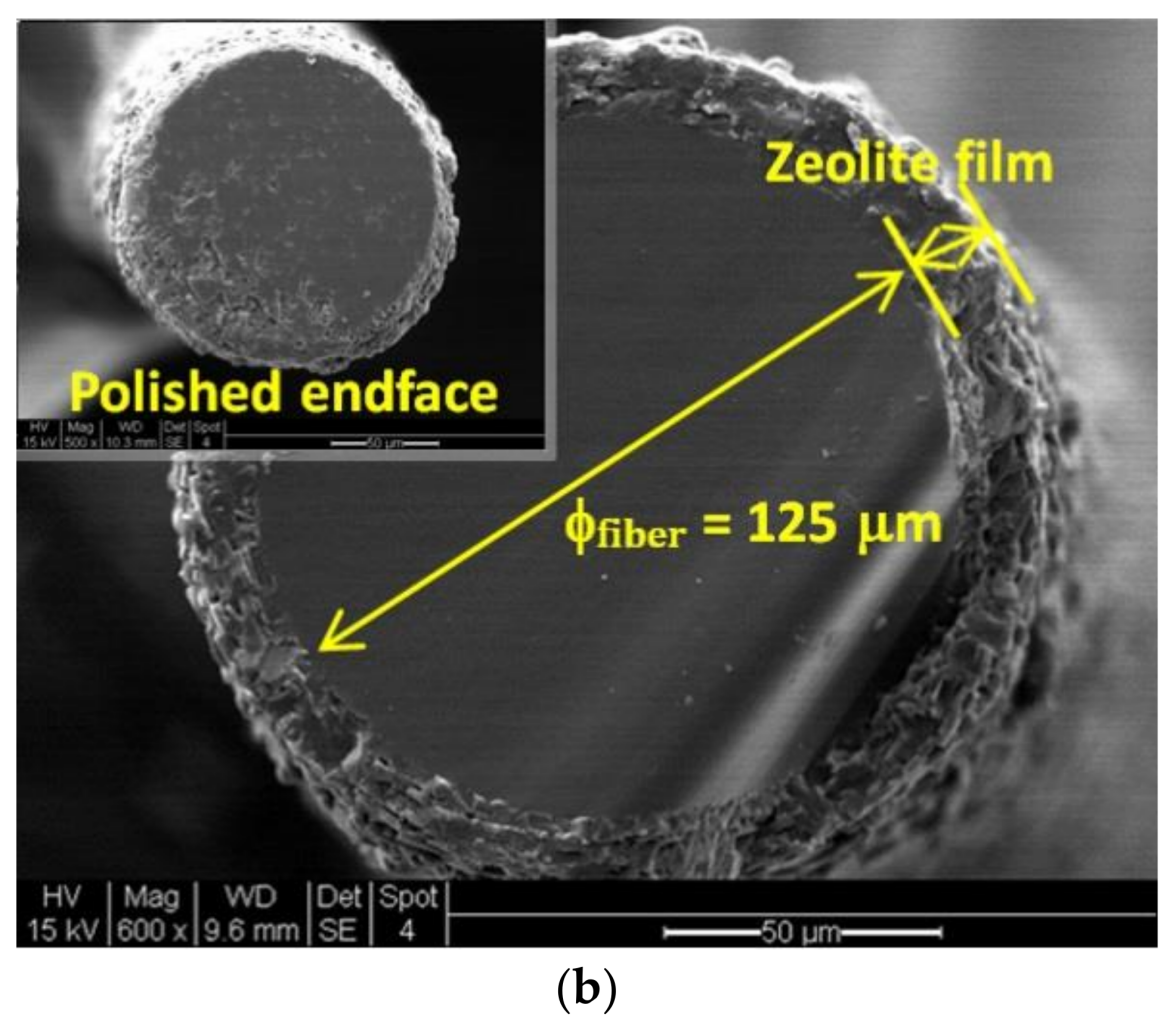
SEM pictures of the zeolite film-coated FOI sensor head: (**a**) unpolished zeolite film on fiber endface; (**b**) fracture cross-section of zeolite coating on the fiber side surface with the insert showing the endface film after polishing.

Figure 4 shows the SEM pictures of the zeolite film-coated FOI sensor head. The film was uniform that generated well-defined sinusoidal interference spectrum after polishing the surface as can be seen later in the paper. The physical thickness of the zeolite film determined from the interferogram in pure helium (or vacuum) was considered as *L_z,_*_0_ because helium (*n* ≈ 1.0) is essentially nonadsorbing in silicalite. The *L_z,_*_0_ was calculated to be 9.8 μm by Equation (4) using the literature value of $n_{z,0}$ (=1.3361 in the near IR region) [8]. This calculated film thickness was consistent with the SEM observation on the film formed on the fiber side surface near the end.

**Figure 5:**
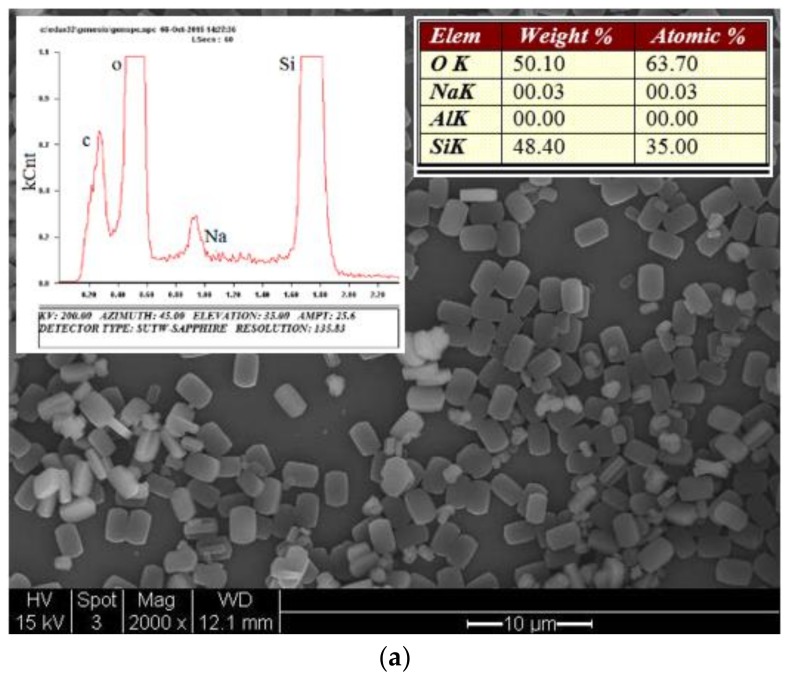

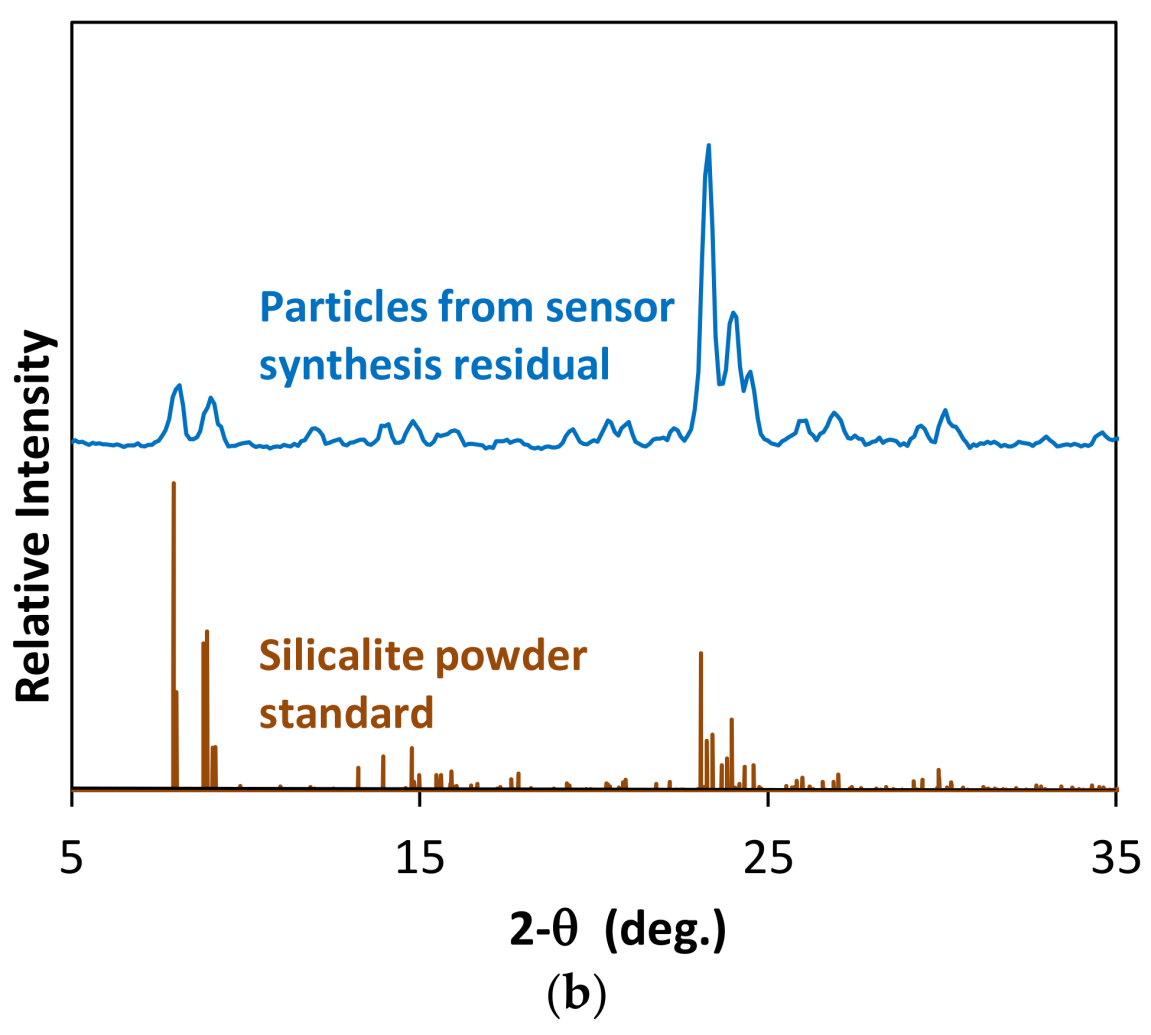
Characterizations of the crystals collected from the residual solution in the zeolite film synthesis vessel: (**a**) SEM picture with inserts showing the EDS spectrum and elemental composition and (**b**) XRD pattern in comparison with standard of silicalite powder.

Zeolite crystals formed in the liquid phase during the hydrothermal crystallization process were collected from the residual in the sensor head (i.e., zeolite film) synthesis vessel. The zeolite particles were cleaned by 0.1 M NaOH solution to remove any unreacted amorphous silica and then rinsed by D.I. water followed by drying at 100 °C in an oven for overnight. This zeolite particle sample was examined by SEM, energy dispersive X-ray spectroscopy (EDS), and X-ray diffraction (XRD) techniques. The results are presented in Figure 5. The individual zeolite crystals had the same shape and morphology as those observed on the surface of the zeolite film formed on the fiber. The EDS elemental analysis revealed that the zeolite framework was essentially of pure silica with no other detectable elements. The very small amount of carbon could be attributed to the TPA^+^ (SDA) molecules in the zeolitic channels and the Na^+^ was likely from the residual of the cleaning solution. The XRD pattern was perfectly consistent with the silicalite powder standard, confirming that the crystals were of pure silicalite phase.

### 3.2. Isobutane Isotherms

**Figure 6:**
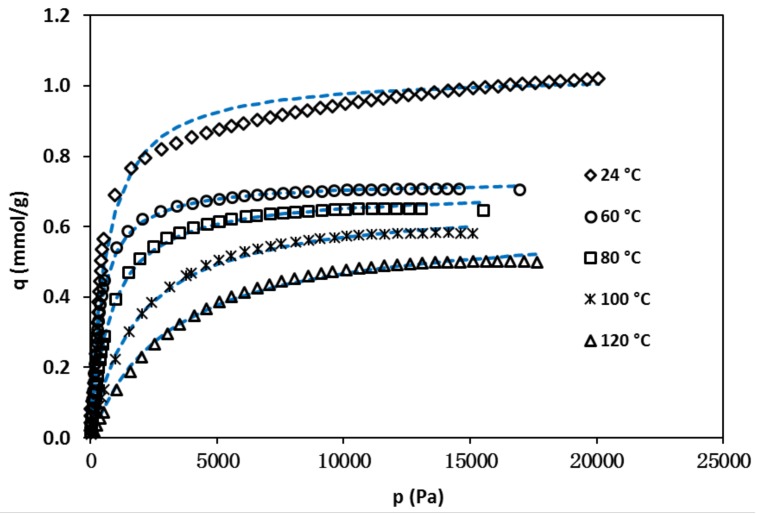
Isotherms of isobutane in silicalite at various temperatures (dashed lines are least square regressions using the extended Langmuir equation).

The isotherms of isobutane measured on the zeolite crystals collected from the liquid phase residual in the sensor synthesis vessel are shown in Figure 6. The isotherms exhibited typical type-1 adsorption behavior that was expected for the microporous zeolites (pore size 0.56 nm). Thus, the Langmuir equation given below was used to described the isotherms,

(8) $$q=q_{\max}\frac{K_{L}p}{1+K_{L}p}$$

where *q* and *q_max_* are amounts of adsorbed gas (mmol/g) at equilibrium gas pressure *p* and maximum amount of adsorption at a given temperature; and *K_L_* is the temperature-dependent Langmuir constant.

The isobutane concentration in zeolite at equilibrium state under gas pressure of *p* is *C_∞_* = *q·ρ_z_* $C=q\cdot \rho_{z}$ and the maximum concentration is $C_{\max}=q_{\max}\cdot \rho_{z}$, where $\rho_{z}$ is the silicalite density (=1.76 g/cm^3^) [18]. The constants $q_{\max}$ and $K_{L}$ were obtained by least-square regression of the isotherms in Figure 6 and their values are listed in Table 1. The Langmuir model provided excellent fitting at temperatures >60 °C but gave relatively large deviations at room temperature likely due to multilayer adsorption at channel intersections under low temperature and high pressure [19]. The diffusivity measurements were conducted at isobutene gas pressure *P_isob_* < 1000 Pa, where the adsorption equilibria are within the Henry’s regions and the Langmuir equation provides excellent representation for all temperatures.

### 3.3. Diffusivity Determination by Optical Measurements

#### 3.3.1. Diffusivity and Concentration Dependence

**Figure 7:**
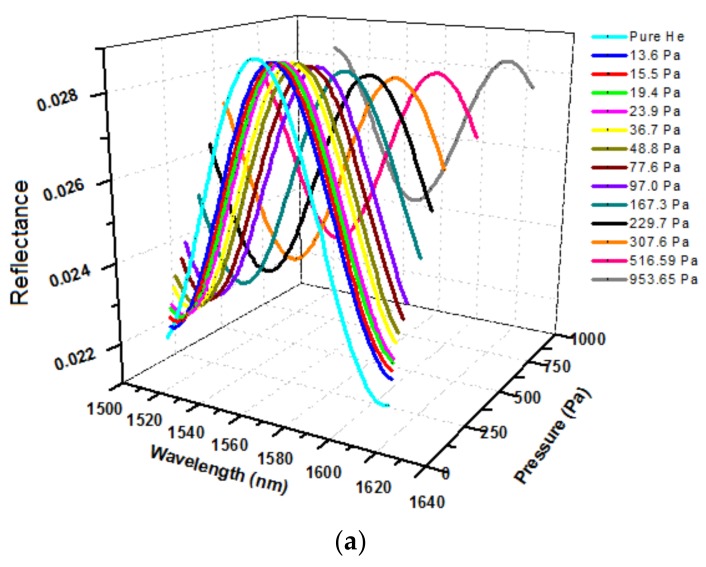

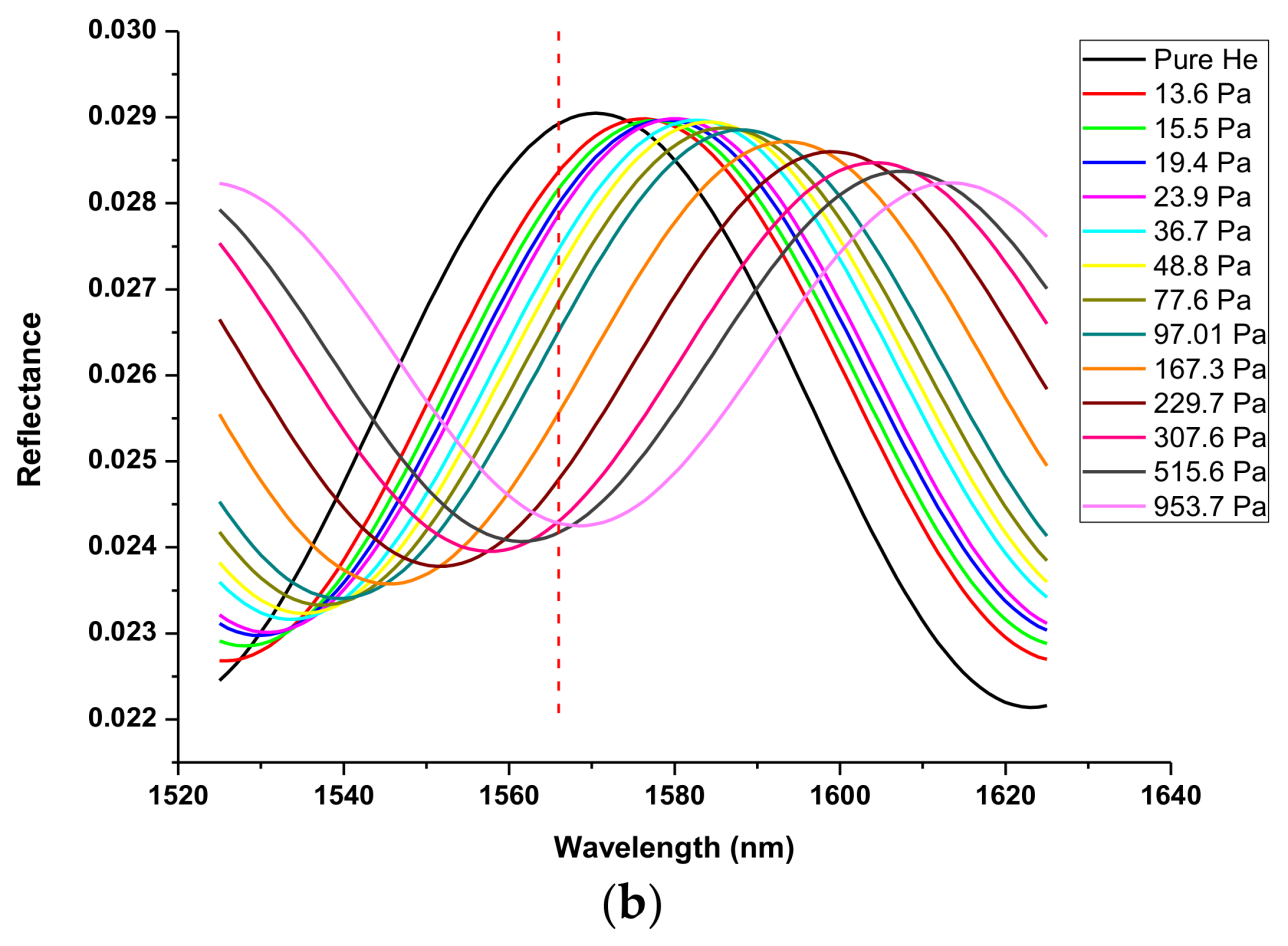
Interferograms of the zeolite film-coated FOI sensor under equilibrium states at various *P_isob_*: (**a**) 3-D presentation and (**b**) 2-D view of interference evolution with equilibrium *P_isob_*.

Experiments were first carried out at room temperature (24 °C) using helium flows carried with isobutane gas of controlled volume fractions or partial pressures. A baseline interferogram was first recorded for the degassed sensor in pure helium flow (100 cm^3^ STP/min) and then the interferogram was determined in gas flows of different isobutane partial pressures (*P_isob_*). The interference spectra recorded at equilibrium *P_isob_* increasing from 0 to 954 Pa are presented in Figure 7. It should be noted that the interferograms at equilibrium states under different *P_isob_* were taken simultaneously with the staircase measurement of the temporal response of single wavelength intensity. When measuring the time-dependent single wavelength intensity for small increments of *P_isob_*, the entire interferogram was also recorded when equilibrium was reached at each *P_isob_* of the staircase. The equilibrium state was indicated by full stabilization of the intensity. Thus, the sensor operation for diffusivity measurement is actually accomplished in one continuous operation. The single wavelength, where monotonic relation between intensity and *P_isob_* exists, can be easily identified based on the baseline interferogram alone.

**Figure 8:**
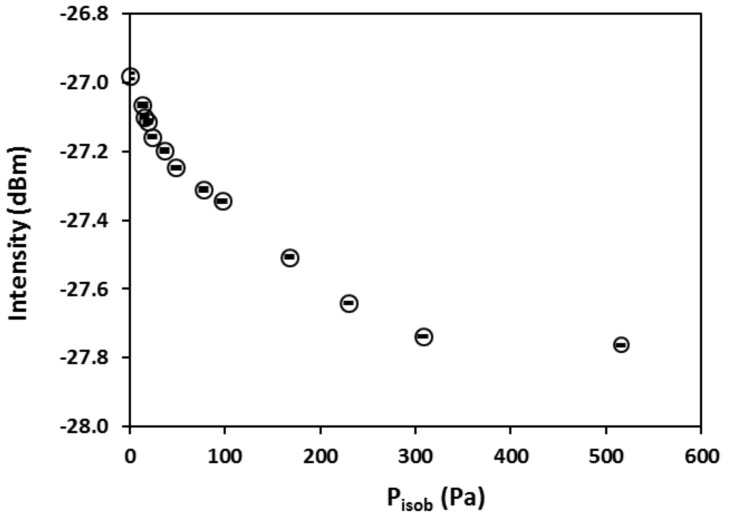
Correlation of the zeolite film-coated FOI reflection intensity at a fixed wavelength of 1566 nm with the *P_isob_* at 24 °C.

As can be seen in Figure 7b, increasing *P_isob_* caused a red shift of the interference spectrum (interferogram) with simultaneous decrease in amplitude. The amplitude decrease was a result of the *n_z_* increase with increasing adsorption that reduces the reflective index contrast at the zeolite/fiber interface, i.e., (*n_f_–n_z_*), where the *n_f_* remains constant, and the shift of spectrum to longer wavelength was a result of increasing optical thickness (*L_z_n_z_*) because the *n_z_* increases and silicalite is known to expand upon adsorbing molecules as well [8]. Figure 7b indicates that the dependence of single wavelength intensity on the amount of adsorption varies with the specific wavelength used. For the current FOI sensor, the intensity at wavelength of 1566 nm exhibits a monotonic dependence on *P_isob_* when *P_isob_* < 1000 Pa. The experimentally measured intensity at λ = 1566 nm is presented in Figure 8 as a function of equilibrium *P_isob_*. The intensity value of each point in Figure 8 was averaged over 30 s after stabilization (i.e., 300 data points) and the standard error bars are shown in the graph. The dependency of reflection intensity on the *P_iosb_*, and hence on the corresponding equilibrium concentration (*C_∞_*), was used later to determine the relationship between the diffusivity and adsorbate concentration.

**Figure 9:**
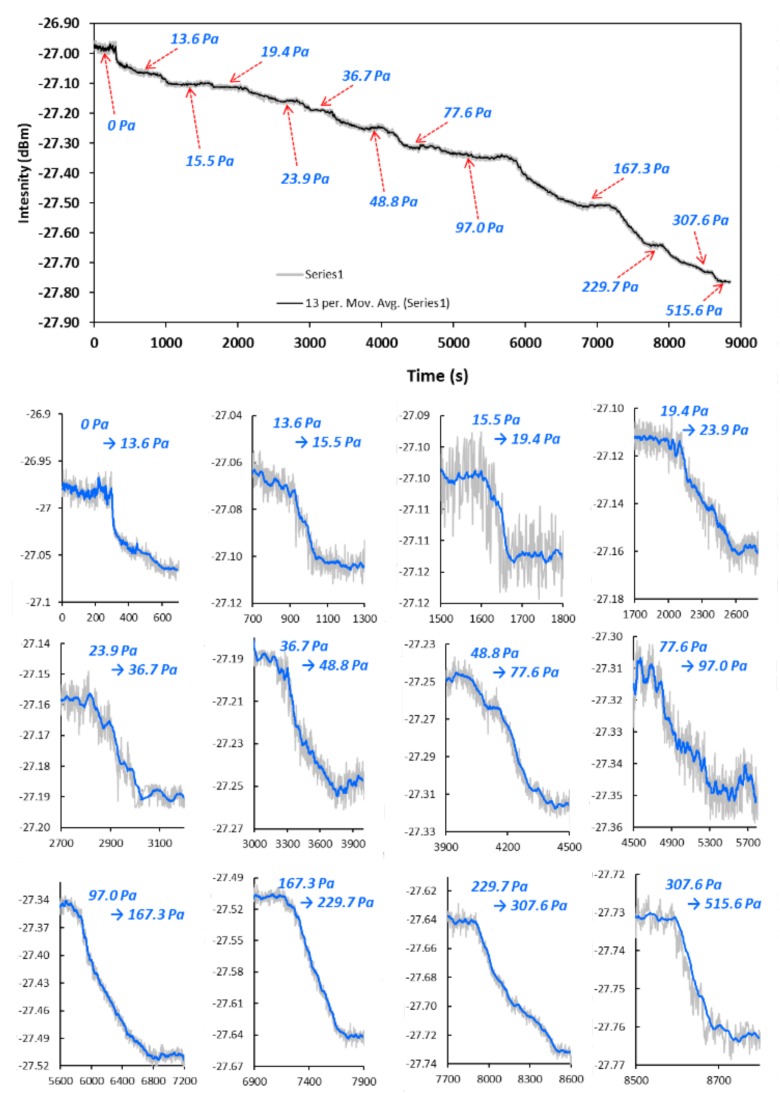
Temporal response of single wavelength (λ = 1566 nm) intensity to staircase variations of *P_isob_* (the top figure shows continuously monitored response and the small figures underneath are temporal response to each of the staircase changes).

**Figure 10:**
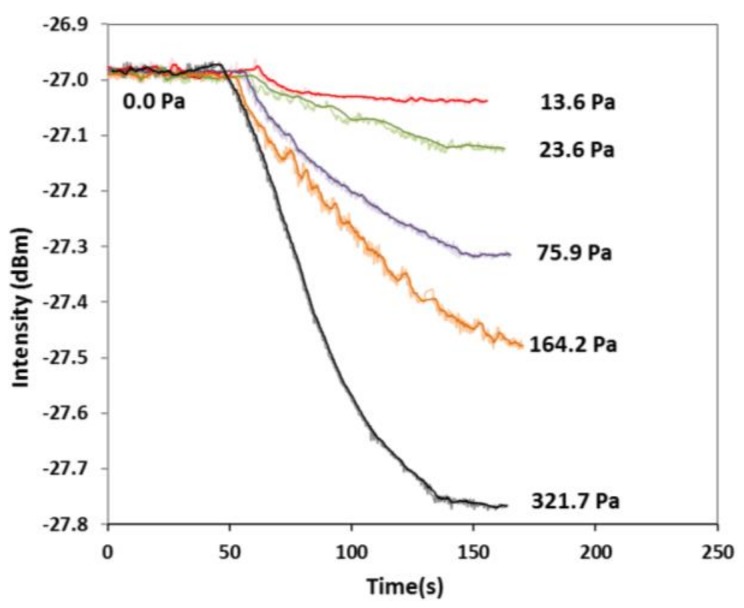
Temporal responses of reflection intensity at λ = 1566 nm to one-step *P_isob_* increase from 0 Pa to various specific values.

FOI to the dynamic process of isobutene adsorption. In the first condition, the measurement of temporal response of single wavelength intensity during a staircase change of *P_isob_* was performed at *λ* = 1566 nm. The results are presented in Figure 9. In the second operating condition, the temporal response of FOI reflection intensity was recorded at *λ* = 1566 nm when the gas phase *P_isob_* was switched from 0 Pa to a specific value without intermediate stairs, and the results are shown in Figure 10. The sensor was completely degassed between measurements of two *P_isob_* values in the second operation condition.

Two types of conditions were used to measure temporal intensity response of the zeolite film-coated. 

**Figure 11:**
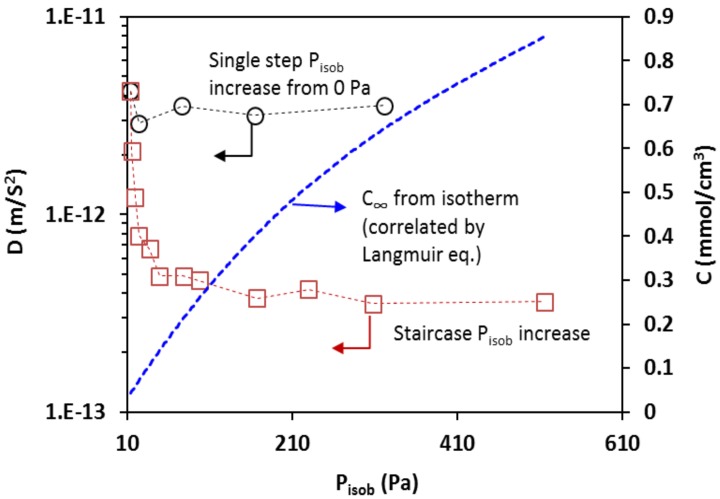
The transport diffusivity measured under the two conditions shown as a function of *P_isob_* together with the corresponding isobutane equilibrium concentration in the zeolite.

With the data of temporal intensity response to *P_isob_* changes, i.e., dynamic changes of *C* in zeolite film, D(C) was computed by Equation (7). Figure 11 presents the transport diffusivity as a function of equilibrium gas pressure *P_isob_* together with the corresponding *C*_∞_. The diffusivity values measured under the second operation condition, i.e., the single step increase of *P_isob_* from 0 Pa, were in range of the literature values obtained at similar temperatures by macroscopic methods such as the MBR, ZLC, concentration pulse CHG, and FTIR spectroscopy techniques. A summary of the isobutane diffusivity values in MFI-type zeolites, including silicalite and ZSM-5, is provided by Table 2. The diffusivity measured under the condition of staircase-increase of *P_isob_* was very close to those measured under the second condition when *P_isob_* was sufficiently low (i.e., <20 Pa) but appeared to be significantly lower at relatively higher pressure. This may be explained by the fact that, when the *P_isob_* was switched directly from 0 Pa to a specific value, the molecular diffusion started in the zeolitic pores free of adsorbate, and the staircase-increase of *P_isob_* involved diffusion in pores that were already filled with adsorbate molecules. In other words, the thermodynamics states of the transporting molecules in the two conditions were different [6]. Thus, the two conditions did not cause large differences at *P_isob_* < 20 Pa where adsorbate loading were low but resulted in significant deviations due to the high concentration of loaded adsorbate at high *P_isob_*. This observation is generally in agreement with the literature findings based on the dual sites adsorption model [19]. In dual site adsorption, adsorbed molecules preferentially reside in the channel intersections of MFI zeolites at low loads; however, at high loading level, diffusivity decreases as the adsorbate molecules inter the cylindrical channel segments (of smaller size than the intersections) and eventually approaches constant values when both types of adsorption sites are highly occupied [20].

#### 3.3.2. Determination of Temperature Dependence

**Figure 12:**
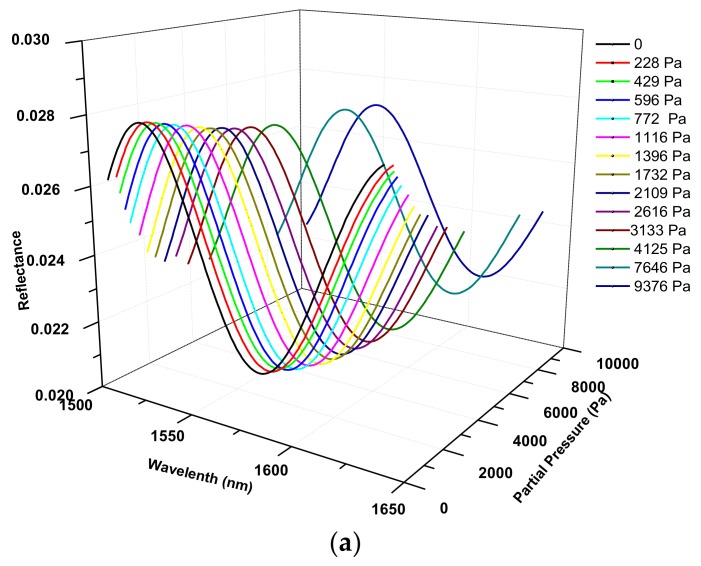

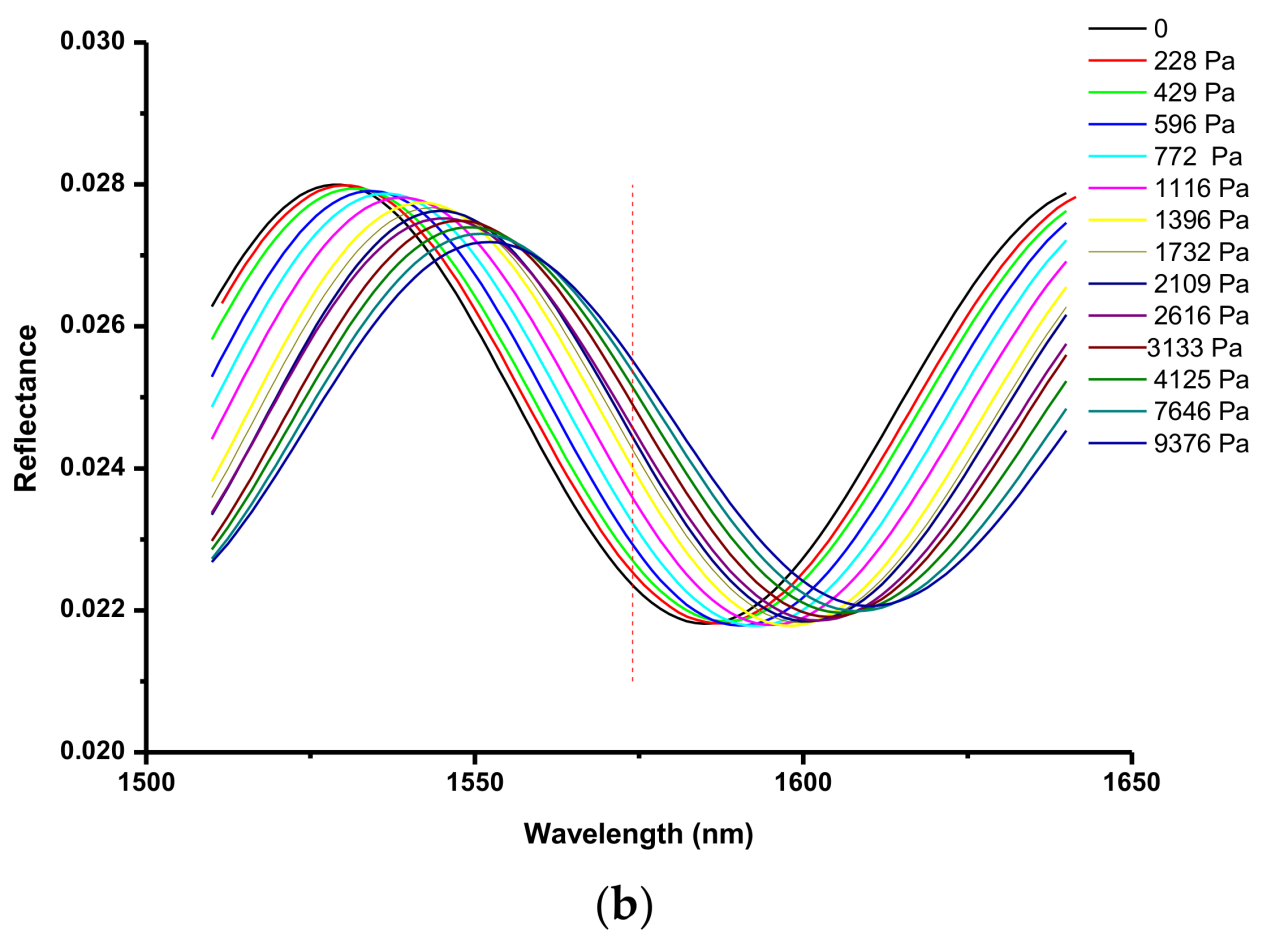
Interference spectra at 80 °C as a function of *P_isob_*: (**a**) 3-D presentation and (**b**) 2-D view of interference evolution with equilibrium *P_isob_*.

**Figure 13:**
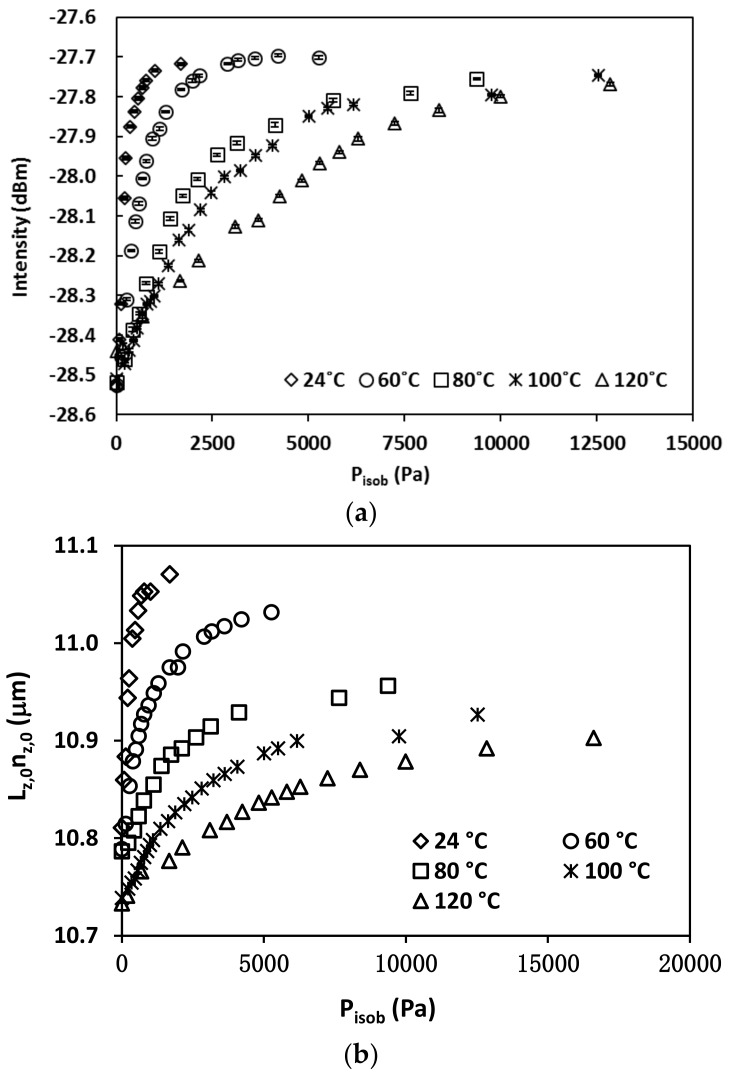
The pressure (*P_isob_*) dependence of (**a**) reflection intensity at a fixed wavelength of 1574 nm, and (**b**) zeolite film optical thickness ($L_{z,0}n_{z,0}$) at various temperatures.

The FOI sensing method was further demonstrated for determining the temperature-dependence of the isobutane diffusivity in the zeolite. In this experiment, the sample gas was provided by the gas tank of preset *P_isob_* without a carrier gas. The interferogram was measured for an identically made FOI sensor, which had a zeolite film thickness *L_z,_*_0_ of 8.06 μm. The thickness of this zeolite film was slightly smaller than the one used earlier most likely due to the variation of the depth of fiber tip immersed in solution during the hydrothermal synthesis. The reflected interference spectra were measured at 24, 60, 80, 100, and 120 °C, respectively. Figure 12 shows an example of the interferogram evolution as a function of *P_isob_* at 80 °C and the spectra for other temperatures are not shown. Because of the slightly thinner zeolite film coating, the single wavelength intensity monitoring was chosen at *λ* = 1574 nm where a monotonically increasing intensity existed at all temperatures. The intensity-dependence on *P_isob_* at all five temperatures are presented in Figure 13. The optical thicknesses of the zeolite film ($L_{z,0}n_{z,0}$) were also calculated from the baseline spectra as discussed before and are provided in Figure 13b. Both the intensity and the change of intensity with *P_isob_* decreased with increasing temperature because the amount of adsorption decreases as temperature increases (see isotherms in Figure 6).

**Figure 14:**
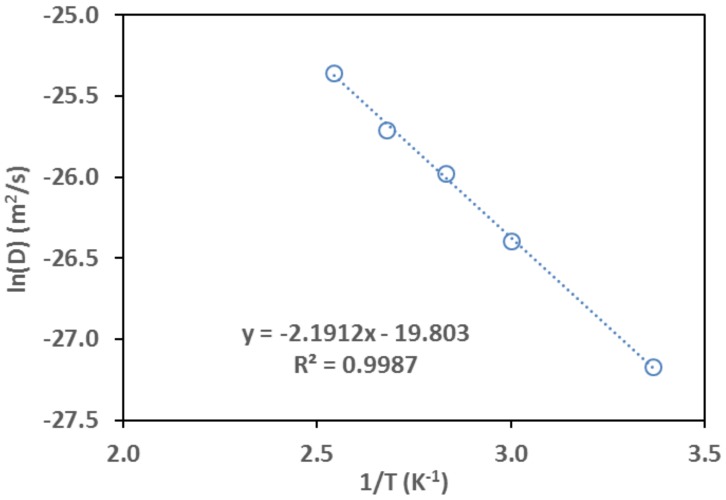
Arrhenius plot of isobutane diffusivity (*D*) obtained by the FOI sensor.

The diffusivity was measured under low pressure (*P_isob_* < 300 Pa) for each temperature using the procedure of single-step change of *P_isob_* from 0 Pa (vacuum after degassing) to the preset value. The diffusivity values are presented by the Arrhenius plot in Figure 14. The calculation of effective diffusion activation energy (*E_d_*) for the following Arrhenius expression was done using the results of linear fitting in Figure 14.

(9) $$D=D_{0,\infty }\exp\left(-\frac{E_{d}}{RT}\right)$$

From the linearly correlated intercept and slope in Figure 14, the $D_{0,\infty }$ and *E_d_* values were found to be 2.51 × 10^−9^ m^2^/s and 18.22 kJ/mol, respectively. The $D_{0,\infty }$ and *E_d_* values found in this work are in reasonable agreement with those reported in the literature obtained by various macroscopic measurement methods as shown in Table 3.

## 4. Conclusions

Zeolite film-coated FOI sensors have been fabricated by directly growing zeolite thin films on the straight-cut endfaces of communication optical fibers. The FOI sensors have shown the capability of measuring molecular transport diffusivity in the coated zeolite films by monitoring the temporal response of the optical interference signals during the dynamic process of gas adsorption. The FOI sensor platform has high sensitivity for responding to ppm-level changes in adsorbate concentration (or adsorbate gas pressure, e.g., <20 Pa for isobutane) that allows for investigating the concentration-dependence of diffusivity via staircase measurement using very small increments in concentration. The effectiveness of this FOI sensor platform for molecular diffusivity measurement has been validated using isobutane as a sample adsorbate gas. The diffusivity of isobutane in the silicalite obtained by the FOI sensing method (e.g., D = 1.2–4.2 × 10^−12^ m^2^/s at 297 K) was in good agreement with literature values obtained by conventional macroscopic techniques such as the MBR and ZLC techniques (e.g., D = 1.1–2.5 × 10^−12^ m^2^/s at 297–303 K). Based on the findings of this work and taking the advantage of high sensitivity of the FOI sensor, our current studies are focused on FOI sensors with films of single layer crystals to investigate the intercrystalline boundary effect on diffusivity measurements, which is a challenging subject in the field. The FOI sensor platform, because of its robustness and small size, could be useful for studying molecular diffusion in zeolitic materials under conditions that are inaccessible to the existing techniques such as high temperature, high pressure, multiphase, and reactive environments. This FOI sensor technology may be improved by employing CCD spectrometers to monitor frequency shift with high speed for measuring zeolites and molecules where large diffusivity values exist. Furthermore, the principle of the diffusivity measurement demonstrated in this study may be applied to the recently developed film-coated endface cavity FOIs [28,29] for determination of molecular and ionic diffusivities in liquid phases.

## Figures and Tables

**Table 1:** Constants for the Langmuir equation correlated from the isobutane isotherms.

*T*, °C	$q_{\max}$, 10^−3^ mol/g	$C_{\max}$, 10^−3^ mol/cm^3^	$K_{L}$, 10^−3^ Pa^−1^
24	1.034	1.8198	1.702
60	0.7309	1.2864	1.562
80	0.7019	1.2353	1.271
100	0.6743	1.1868	0.541
120	0.6143	1.0811	0.317

**Table 2:** Summary of isobutane diffusivity at low *P_isob_* in comparison with literature values measured by macroscopic methods under comparable conditions.

Zeolite	*T*, K	*D*, 10^−12^ m^2^/s	Method	Source
Silicalite	297	1.20–4.20	FOI (1)	This work
Silicalite	297	2.89–4.20	FOI (2)	This work
Silicalite	297	1.90	MBR	[21]
Silicalite	303	2.50	MBR	[22]
Silicalite	303	1.10	MBR	[23]
Silicalite	298	1.37–2.29	FTIR	[7]
Silicalite	303	1.49	ZLC	[24]
ZSM-5 (Si/Al = 36)	303	7.00	MBR	[25]
ZSM-5 (Si/Al = 35)	293	0.14	ZLC	[26]

(1) and (2) are conditions of staircase and one-step pressure increase, respectively, at *P_isob_* < 50 Pa.

**Table 3:** Comparison of apparent activation energy of diffusion with literature values.

Zeolite	$D_{0,\infty }$, 10^−9^ m^2^/s	*E_d_*, kJ/mol	Method	Source
Silicalite	2.51	18.22	FOI	this study
Silicalite	4.30	32.2	ZLC	[24]
Silicalite	3.29	24.3	ZLC	[26]
ZSM-5 (Si/Al = 35)	6.70	31.6	MBR	[25]
ZSM-5 (Si/Al = 36)	1.50	15.1	MBR	[22]
ZSM-5 (Si/Al = 36)	NA	24.2	CHG	[27]
